# NERVE GROWTH FACTOR, NEUROPEPTIDES AND CUTANEOUS NERVES IN ATOPIC DERMATITIS

**DOI:** 10.4103/0019-5154.62735

**Published:** 2010

**Authors:** Abeer Hodeib, Zeinab Abd El-Samad, Hesham Hanafy, Amani Abd El-Latief, Amal El-bendary, Azza Abu-Raya

**Affiliations:** *Departement of Dermatology and and Venereology, Tanta University, Egypt.*; 1 *Departement of Clinical Pathology, Tanta University, Egypt.*; 2 *Departement of Histology, Tanta University, Egypt.*

**Keywords:** *Atopic dermatitis*, *nerve growth factor*, *neuropeptides*

## Abstract

**Introduction:**

Neurogenic components, as neurotrophic factors and neuropeptides, are probably involved in the pathogenesis of atopic dermatitis (AD) with the neuroimmunocutaneous system as they modify the functions of immunoactive cells in the skin. Nerve growth factor (NGF) is the best-characterized member of the neurotrophin family. Both NGF and neuropeptides (NPs) may be associated with the disease pathogenesis.

**Aim:**

This study aims to evaluate the plasma level of NGF and NPs in AD patients and correlate them with the disease activity and nerve changes in the skin by electron microscopy.

**Materials and Methods:**

Plasma levels of NGF and vasoactive intestinal peptide (+VIP) were measured by an immunoenzymatic assay while plasma levels of calcitonine gene related peptide (CGRP) and neuropeptide Y (NPY) were measured by radioimmunoassay in 30 AD patients in comparison to 10 normal non-atopic controls. Electron microscopic study was done in 10 AD patients.

**Results:**

It has been found that there is significant increase of plasma levels of NGF and NPs in AD patients compared with controls. There is a positive correlation between the plasma levels of NGF and disease activity (correlation coefficient = 0.750, *P*<0.005). There is a significant correlation between the number of Schwann axon complex, evidenced by electron microscopic examination and plasma level of NGF in AD patients.

**Conclusion:**

It has been concluded that these neurogenic factors; NGF and NPs modulate the allergic response in AD, probably through interactions with cells of the immune-inflammatory component. NGF might be considered as a marker of the disease activity.

## Introduction

The pathogenesis of atopic dermatitis (AD) is multifactorial, involving genetic, psychological, and immunological factors. There is considerable evidence that suggests that the nervous system can influence the course of the disease through emotional stress, altered patterns of cutaneous innervation and abnormal expression of neuropeptides (NPs) in lesional skin. Neuropeptides are produced and released mainly by the peripheral nervous system, and may be by both the immune and the endothelial system.[1] NPs play a role in immediate and delayed-type hypersensitivities as well as in neurogenic inflammation suggesting their possible involvement in the pathogenesis of AD.[2]

Tissue levels of NPs have been reported to be increased in chronic lichenified lesions of AD,[3,4] but a few studies were done on the serum level of NPs in AD and their correlation with the disease activity.[5,6] Nerve growth factor (NGF) is the best-characterized member of the neurotrophin family. It is essential for the survival, development, differentiation and function of peripheral sympathetic, sensory neurons and basal forebrain cholinergic neurons in the central nervous system.[6] NGF also acts as a neurotrophic molecule in the skin. As it stimulates the sprouting of the nerve fibers and modulates the synthesis and expression of NPs, it is considered to be a primary candidate as a regulatory molecule in neurogenic responses.[7]

This study was done to gain further insight into the systemic involvement of neurogenic factors in association with the immune system during the pathogenesis of AD. We measured the plasma levels of NGF, vasoactive intestinal peptide (VIP); calcitonin gene related peptide (CGRP) and neuropeptide Y (NP-Y) in AD patients and correlated their levels with the disease activity. Ultra structural examination of cutaneous nerves in 10 AD patients was performed to correlate the results with serum levels of NGF and NPs.

## Materials and Methods

The study was done on 30 AD patients (16 men, 14 women; mean age 25 years, range 16-41), with a confirmed diagnosis of AD according to the criteria of Hanifin and Rajka.[8] None of the patients received any topical treatment for at least two weeks or systemic treatment one month prior to the study. Ten healthy subjects (five men, five women; mean age 26 years, range 19-38) with no history of atopic diseases served as the control group. None of the subjects had any other concomitant dermatological or medical disorders.

The patient group was subdivided into three subgroups according to their skin condition as determined by the six area six sign atopic dermatitis (SASSAD) severity score[9]; a simple and effective system for recording and monitoring AD activity. It is obtained by grading six signs (erythema, exudation, excoriation, dryness, cracking and lichenification), each on a scale of 0 (absent), one (mild), two (moderate), or three (severe), at each of six sites; arms, hands, legs, feet, head and neck, trunk.

### The following were done for all patients and controls

- Venous peripheral blood sample was centrifuged and the plasma was stored at -70°C until assayed for NGF and NPs as follow: NGF was measured using a highly sensitive ELISA that recognizes human and murine NGF using a commercial kit, following a modification of the protocol described by Boehringer Mannheim (Mannheim Germany).[10]
- Vasoactive intestinal peptide was measured using specific ELISA kits (Peninsula laboratories Ins. San Carlos CA) according to the manufacture protocol.[5]
- Human CGRP was determined by competitive radioimmunoassay according to the described protocol.[11]
- Neuropeptide Y was determined by competitive radioimmunoassay using an antiserum raised against synthetic NPY conjugated to borin thyroglobin produced by DRG (USA) and I (125) labeled NP-Y.[12]

#### Electron microscopy

Under local anesthesia, 4mm punch biopsies were obtained from the arms of 5 moderate AD patients, 5 severe AD patients, and 5 control subjects. The specimens were immediately processed and prepared for electron microscopic examination.[13]

## Results

Using SASSAD severity score, it was found that 10 patients had mild skin changes (score <21), 10 patients had moderate skin changes (score 21-40), and 10 patients had severe skin changes (score >41). Circulating NGF levels in AD were significantly increased compared with healthy controls (2510.5±572 pg/ml vs. 89.8±55.9 pg/ml respectively, *P*<0.0005). Circulating VIP levels in patients with AD were significantly increased compared with healthy controls (345.8±71.5 μg/ml vs. 307.1±42.6 μg/ml respectively. *P*<0.05). The mean serum level of CGRP in all AD patients was (133.7±28.6) pg/ml. The value was significantly higher than that in controls (82.2±9.1) pg/ml (*P*<0.05). The mean serum level of NP-Y in all AD patients was (54.314±9.91) P mol/L. The value was significantly higher than that in controls (11.61±7.40) P mol/L (*P*<0.05) [Table 1].

**Table 1:** Serum levels of NGF and NPs in AD patients compared to control

Neuropeptide	Control	Atopic dermatitis patients	*P*
NGF (pg/ml)	89.8±55.9	2510.5±572	<0.0005
VIP (μg/ml)	307.1±42.6	345.8±71.5	<0.05
CGRP (pg/ml)	82.2±9.1	133.7±28.6	<0.05
NPY (P mol/L)	11.61±7.40	54.314±9.91	<0.05

By comparing the levels of NGF and NPs in each group of AD, it was found that, NGF was significantly increased in all AD groups, VIP and CGRP were significantly elevated in moderate group, while NPY was elevated in moderate and severe groups of AD as compared to controls [Table 2].

**Table 2:** Serum levels of NGF and NPs in different subgroups of AD patients compared to control

NPs	Mild AD	Moderate AD	Severe AD	Control	*P*
NGF	1970±230	2199±318	3297±412	89.8±55.9	<0.05*
VIP	345±65	321±70	429±62	307.1±42.6	>0.05
CGRP	110±23	115±52	170±85	82.2±9.1	>0.05
NPY	34.7±15	43.9±9.8	72.9±11.9	11.61±7.4	>0.05

### 

#### Correlation of neuropeptides with SASSAD severity score

The serum level of VIP, CGRP and NPY were found to be not correlated directly with degree of severity of AD measured by SASSAD score (*P*>0.05). The serum level of NGF was found to be correlated directly with degree of severity of AD measured by SASSAD score (*P*<0.005, r = 0.750).

#### Results of ultra structural study

**Figure 1:**
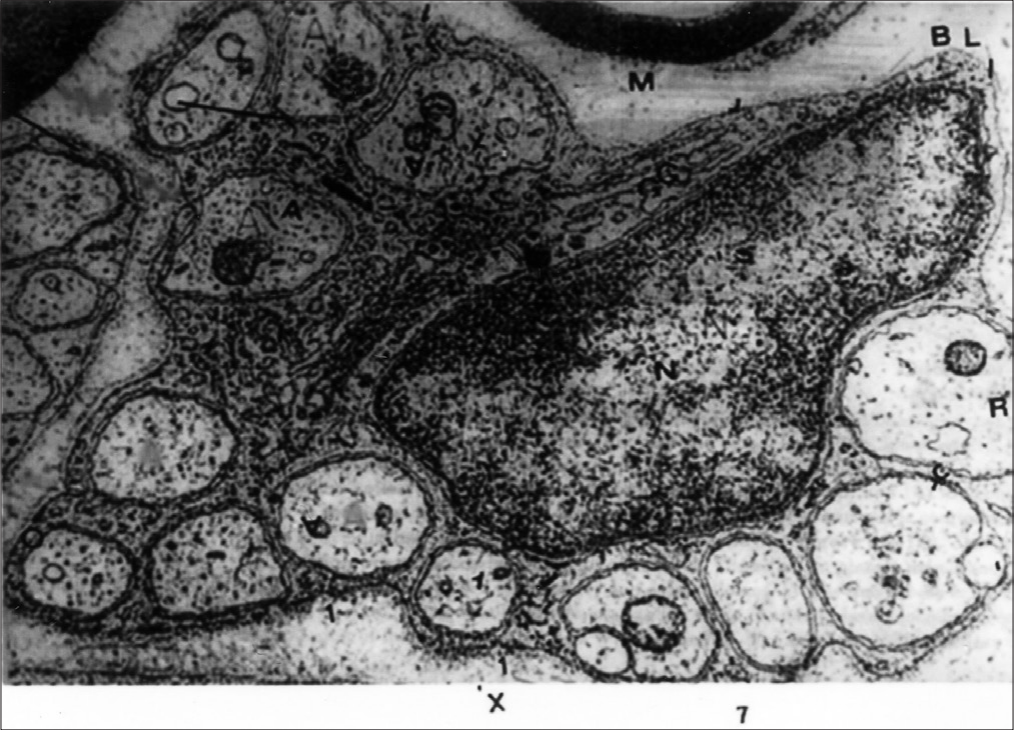
Unmyelinated nerve fibers of the dermis in AD. The individual fibers or axons (A) are embedded in cytoplasm of Schwann cell. The arrows indicate the site of the mesaxon which is enclosed by schwann cell cytoplasm. Evident nucleus (N). The golgi apparatus (G) and their surrounding basal lamina (BL). A myelinated nerve fiber is seen in upper part (EM ×26000)

**Figure 2:**
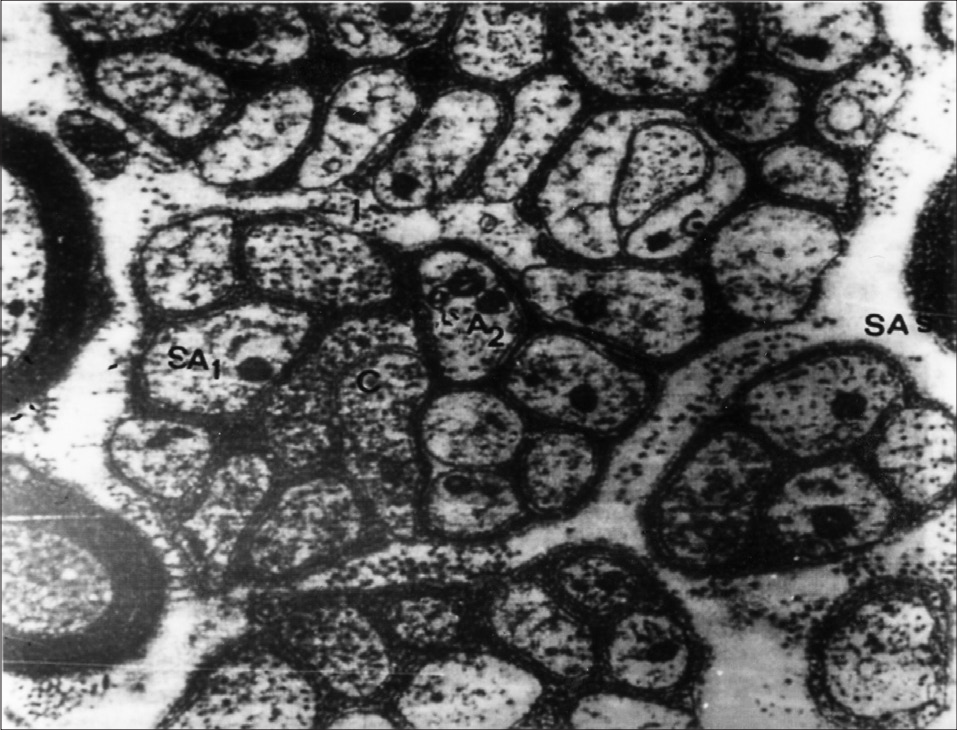
EM of AD showing many nerve fascicles having many Schwann cell axon complexes (SAs) which are either mono (SA1) or oligo (SA2) nerve fibers (EM ×8000)

**Figure 3:**
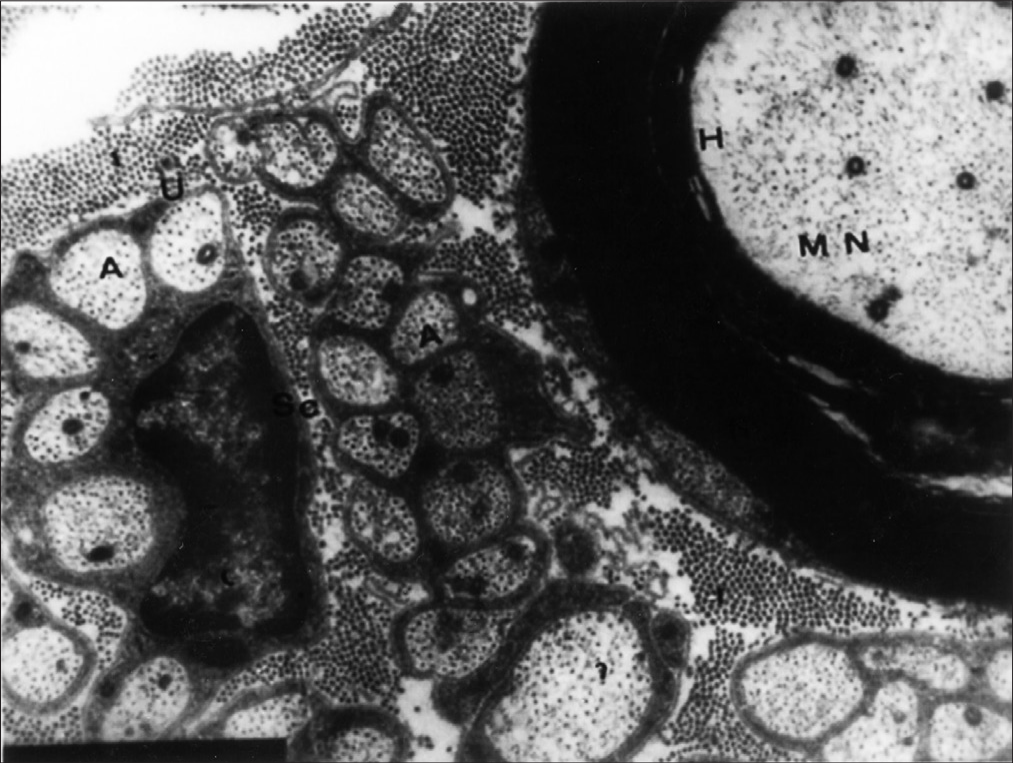
Unmyelinated dermal nerve fibers from control showing axons (A) surrounded by cytoplasm of Schwann cell (SC). A nearby myelinated nerve is seen in upper left quadrant (MN) (EM ×8000)

Most of the cutaneous nerve fibers observed on TEM were unmyelinated. In the dermis, each nerve fascicle with many Schwann cell-axon complexes (SAs) was unsheathed completely by the perineurium and endoneurium in both the lesion-bearing skin and the control [Figure 1]. As the nerve fascicles came to lie near the epidermis, they were partially ensheathed by the perineurium and endoneurium. However, the number of the SAs in the nerve fascicles appeared to be much greater in AD skin than in the controls [Figures 2 and 3].

**Figure 4:**
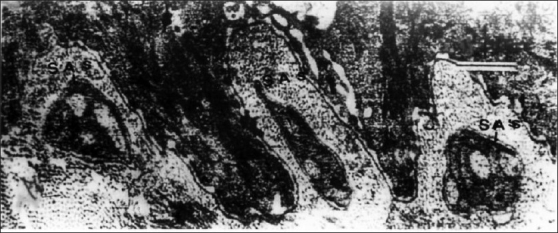
EM of AD showing every dermal micropapilla has one subepidermal SAs that are mostly polyaxinal (EM ×10000)

**Figure 5:**
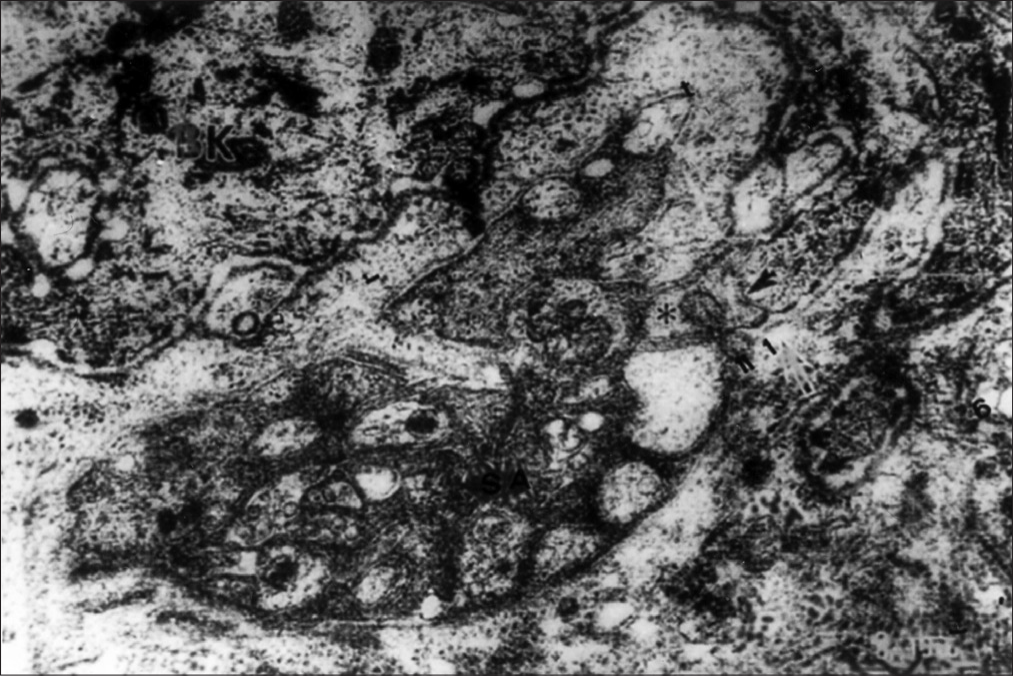
An intraepidermal SA has many axons, one which contact with the tip of microrete ridges (↑). Double arrows (↑↑) indicate the fusing portion between the basal lamina of SA and that of basal keratinocytes (EM ×20000)

In the subepidermal portion, in contrast to the controls in which there were only occasionally SAs in the dermal micropapillae, every dermal micropapilla in the AD skin had one SA in the same areas [Figure 4] and SAs were grouped in the remaining areas [Figure 2]. These axons were completely or partially ensheathed by Schwann sheaths [Figures 1 and 4]. Unlike the normal controls, AD specimens exhibited the nucleated and non-nucleated SAs coexisting next to each other immediately beneath the epidermis [Figure 4]. In the epidermis of AD, the SAs were found in the intercellular spaces between the basal cells and axons, making direct contact with the cell membrane of the basal keratinocyte [Figure 5]. The SA had basal lamina in a relatively wide space between the SA and the surrounding keratinocyte.

**Figure 6:**
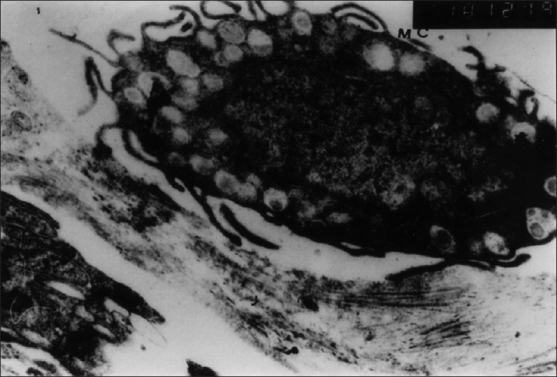
EM of mast cell (MC) from lesional dermis of AD showing evident degranulation. The granules from amorphous bodies (EM ×10000)

Intraepidermal nerve fibers were not observed in the controls. In AD, the axons of the Schwann cells in the subepidermal and intraepidermal SAs generally showed no pathological changes. Mast cells were encountered much more often in the AD skin in contact to nerves and showed evident degranulation than in the controls [Figure 6].

The mean value of the number of the SAs per 100 basal cells was 10.14±7.5 in AD lesions and 1.11±1.02 in the normal control. The difference was statistically significant (U = 0, *P* = 0.0038).

The mean diameter of the SAs was 1.14±0.52 μm in the lesions of AD patients, and 1.19±0.48 μm in the skin of the normal controls. The difference was statistically significant (*P* = 0.0320). The mean number of the axons was 6.48±1.88 in the lesional skin, and 2.52±0.80 in the normal controls. The difference was statistically significant (*P* = 0.0315). The mean diameter of the axons was 0.60±0.08 μm in the lesions of AD patients, and 0.36±0.14 μm in the skin of the normal controls. The difference was statistically significant (*P* = 0.0037). On evaluation of the inter-relationship between the diameter and the number of the axons in the SAs, a Spearman's rank correlation coefficient of r = 0.556, indicative of a positive correlation, was obtained. This indicates that larger diameter of the SAs in the lesion-bearing skin specimens resulted from an increased number of axons in each SA.

Correlation of the serum level of NGF with the number of Schwann cell-axon complexes was indicative of positive correlation (*P*<0.05, r = 0.646).

## Discussion

The study clearly demonstrates that:

1. Patients with AD have significant increases in their plasma levels of NGF and NPs.
2. NGF level in AD patients significantly correlates with the disease severity and with increased number of SAs complex in the skin as evidenced by electron microscopy.
3. Electron microscopy demonstrated increased number of cutaneous nerves in AD patients without damage.

These findings suggest the systemic involvement of neurogenic factors in AD and the significant role of NGF and NPs in the pathogenesis of AD. In addition, they may modulate the allergic response of this disease, probably through interactions with multiple target cells of the immune system. At present, the functional role and the major sources of increased NGF in the plasma of AD patients are unknown and still highly speculative. There is considerable evidence that NGF is able to exert a variety of effects on cells of the immune system. NGF accumulates at the site of inflammation displaying a potent chemotactic for polymorphonuclear leucocytes[14] and inducing activation of eosinophils.[15] On the other hand, proinflammatory cytokines, such as tumor necrosis factor α and interleukin 1, may vigorously stimulate NGF production, leading to an early and maintained increased NGF during inflammation, which might result in an enhanced recruitment of inflammatory cells in the affected tissues.[16,17] The observation that mast cells and T-helper (Th2) lymphocytes express TrkA, a high-affinity tyrosine kinase receptor for NGF, which is able to synthesize and release NGF, along with evidence that NGF acts on other immune cells that are characteristic of allergic inflammation, such as basophils and eosinophils suggest that NGF may be involved in human allergic diseases such as AD.[18]

Moreover, it is suggested that NGF stimulates T-lymphocytes proliferation and induces mast cell proliferation, activation and degranulation. AD is characterized by Th2 cell predominance and increased number of mast cells in the dermis. Therefore, it was postulated that increased plasma level of NGF in AD could be partly derived from the enhancement, activation and proliferation Th2 cells and/or mast cells, which are able to synthesize, store and release NGF.[10]

NGF is also synthesized and released by human keratinocytes. It is postulated that keratinocytes in severe AD might release a greater amount of NGF, which in turn may contribute to the subsequent increase in plasma levels of NGF in parallel to the disease severity.[19]

The present study demonstrates a positive correlation between serum level of NGF and disease severity detected by SASSAD score. This agreed with previous study of Toyoda *et al*. 2002 that confirmed that NGF level correlates with the AD severity measured by two different scores; the objective severity score (SCORAD) and the Eczema Area and severity index (EASI).[10] They suggest that NGF systemically modulates the allergic response in AD, probably through interactions with cells of the immune-inflammatory component.

Regarding NP levels, the present study demonstrated elevated level of NPs in AD patients but not correlated with the disease severity, which agree with the previous studies.[5] The source of elevated serum NPs in AD is still unknown. It has already been known that NP levels in the lesional skin of AD patients were increased,[3,4] together with increased nerve contents in AD skin; it could be concluded that serum NPs in AD might be derived from nerve endings in the skin.

In the immune system, two sources for NPs have been described, (1) the nerve terminals present in central and peripheral lymphoid organs such as thymus, spleen and lymph nodes,[19] and (2) the inflammatory and immune cells such as lymphocytes, mast cells, eosinophils and neutrophils.[20] It has been reported that Th1 cells do not produce VIP, and only Th2 cells produce VIP, raising the possibility that VIP is a Th2 cytokine. Therefore it was speculated that nerves in the skin and Th2 lymphocytes could be possible sources for the increased VIP in AD.[21]

The exact function of NPs is still unknown. It is supposed that CGRP and NPY may have a vasodilator effect that modulates some macrophages functions, including antigen presentation. CGRP is commonly associated with Langerhans cells and with Merkel cells in the epidermis supporting its immunomodulatory effects.[22] VIP carries out many important physiologic functions including vasodilatation, mast cell degranulation, immunomodulation, production of cAMP and cytokines and cell proliferation in keratinocytes.[23]

Electron microscopy in the present study demonstrated increased density of cutaneous nerve fibers in AD, but with no damage which indicates that, they may have function. This agrees with other reports.[24,25] Raap *et al*. 2005[26] found prominent increase of neuropeptides and neuropeptide-positive nerve fibers in lesions of AD. They reported that the density of nerve fibers increased while peripheral nerve endings are in an active state of excitation. Moreover, the dermal contacts between mast cells and nerves were increased in number. This agrees with Jarvikallia *et al*. 2003[27] who suggested that the increased number of mast cells in AD lesions are able to maintain neurogenic inflammation through activation by NPs released from the epidermal nerves. These NPs may also stimulate keratinocytes to release cytokines which affect various cell types enhancing inflammation. The positive correlation of NGF with number of SAs in the skin indicates that the cutaneous nerves, in AD, may be a source for increased NGF level in the serum.

It is speculated that as proper understanding of interactions between the cutaneous neurosensory system and various components of skin and immune system in health and disease increases, specific treatment modulating the neurocutaneous system will find their way into the armamentarium of daily dermatologic therapy.
